# Ovarian cancer relies on the PDGFRβ–fibronectin axis for tumorsphere formation and metastatic spread

**DOI:** 10.1002/1878-0261.13556

**Published:** 2023-12-01

**Authors:** Núria Gendrau‐Sanclemente, Agnès Figueras, Kristina Gracova, Álvaro Lahiguera, Elisenda Alsina‐Sanchís, Juan A. Marín‐Jiménez, August Vidal, Xavier Matias‐Guiu, Sergi Fernandez‐Gonzalez, Marc Barahona, Lola Martí, Jordi Ponce, Francesc Viñals

**Affiliations:** ^1^ Program Against Cancer Therapeutic Resistance (ProCURE) Catalan Institute of Oncology (ICO), Hospital Duran i Reynals Barcelona Spain; ^2^ Oncobell Program Institut d'Investigació Biomèdica de Bellvitge (IDIBELL) Barcelona Spain; ^3^ Cancer Immunotherapy (CIT) Group‐ProCURE Bellvitge Biomedical Research Institute (IDIBELL) – OncoBell Barcelona Spain; ^4^ Department of Medical Oncology Catalan Institute of Oncology (ICO) Barcelona Spain; ^5^ Department of Pathology University Hospital of Bellvitge (IDIBELL) Barcelona Spain; ^6^ CIBERONC Instituto de Salud Carlos III Madrid Spain; ^7^ Department of Gynaecology University Hospital of Bellvitge (IDIBELL) Barcelona Spain; ^8^ Departament de Ciències Fisiològiques Universitat de Barcelona Spain

**Keywords:** fibronectin, HGSOC, metastasis, ovarian cancer, PDGFRβ, tumorspheres

## Abstract

High‐grade serous ovarian cancer (HGSOC) is the deadliest gynecological malignancy. The most common form of metastatic spread of HGSOC is transcoelomic dissemination. In this process, detached cells from the primary tumor aggregate as tumorspheres and promote the accumulation of peritoneal ascites. This represents an early event in HGSOC development and is indicative of poor prognosis. In this study, based on tumorspheres isolated from ascitic liquid samples from HGSOC patients, ovarian cancer spheroid 3D cultures, and *in vivo* models, we describe a key signal for tumorsphere formation in HGSOC. We report that platelet‐derived growth factor receptor beta (PDGFRβ) is essential for fibronectin‐mediated cell clustering of ovarian cancer cells into tumorspheres. This effect is mediated by the kinase NUAK family SNF1‐like kinase 1 (NUAK1) and blocked by PDGFRβ pharmacological or genetic inhibition. In the absence of PDGFRβ, ovarian cancer cells can be provided with fibronectin by cancer‐associated fibroblasts to generate chimeric spheroids. This work provides new insights that uncover potential targets to prevent peritoneal dissemination, the main cause of advanced disease in HGSOC patients.

## Graphical abstract

In this study, we describe a key signal for tumorsphere formation in high‐grade serous ovarian cancer (HGSOC). We report that platelet‐derived growth factor receptor beta (PDGFRβ) is essential for fibronectin‐mediated cell aggregation of ovarian cancer cells into tumorspheres. In the absence of PDGFRβ, ovarian cancer cells can be provided with fibronectin by cancer‐associated fibroblasts (CAFs) to generate chimeric clusters.

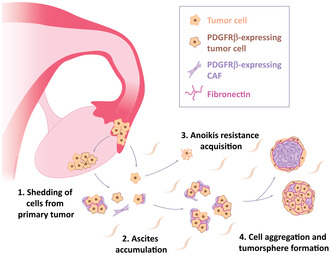

**Abbreviations**
AOI: area of interest
BCA: bicinchoninic acid
bFGF: basic fibroblast growth factor
BSA: bovine serum albumin
CAF: cancer‐associated fibroblast
CCDP: cisplatin
CRC: colorectal cancer
CRC‐LM: colorectal cancer liver metastasis
DAPI: 6‐diamidino‐2‐phenylindole
DMEM: Dulbecco's modified Eagle medium
ECM: extracellular matrix
EGF: epidermal growth factor
EOC: epithelial ovarian cancer
FBS: fetal bovine serum
FFPE: formalin‐fixed paraffin‐embedded
FIGO: International Federation of Gynecology and Obstetrics
FN: fibronectin
Gefi: gefitinib
GEO: Gene Expression Omnibus
HGSOC: high‐grade serous ovarian cancer
iNUAK1: NUAK family SNF1‐like kinase 1 inhibitor
Lapa: lapatinib
LKB1: liver kinase B1
MSCs: mesenchymal stromal stem cells
NSG: NOD‐scid IL2Rgamma null mice
NUAK1: NUAK family SNF1‐like kinase 1
Pazo: pazopanib
PBS: phosphate‐buffered saline
PCR: polymerase chain reaction
PDGFRB‐OE: overexpression of PDGFRB
PDGFRα: platelet‐derived growth factor receptor alpha
PDGFRβ: platelet‐derived growth factor receptor beta
RBC: red blood cell
ROI: region of interest
RT: room temperature
RT‐PCR: real‐time polymerase chain reaction
sh‐CTRL: shRNA empty vector
sh‐CTRL‐Luc: shRNA empty vector and luciferase‐expressing
sh‐PDGFRB: shRNA‐silenced for PDGFRB
sh‐PDGFRB‐Luc: shRNA‐silenced for PDGFRB and luciferase‐expressing
shRNA: short‐hairpin RNA
Suni: sunitinib
TBS: Tris‐buffered saline
TGFβRi: TGFβ receptor inhibitor

## 1 Introduction

Epithelial ovarian cancer (EOC) ranks as the fifth‐leading cause of cancer death among women and represents the most lethal gynecological malignancy [1]. Among the different subtypes, high‐grade serous ovarian cancer (HGSOC) is the most prevalent and aggressive one [2]. HGSOC accounts for 70% of ovarian cancer deaths [1, 3] and < 50% of patients survive beyond 5 years [4]. This is mainly due to the fact that ~ 80% of patients are diagnosed at advanced stage, when the tumor has already disseminated beyond the pelvic region (FIGO stages III–IV) [5, 6], and to the lack of effective therapies for advanced‐stage disease [7]. Peritoneal implants and ascites accumulation are the most common presentations in advanced HGSOC patients [8]. In fact, more than one‐third of women with ovarian cancer will develop ascites during the course of their disease [9] and nearly all present with it at the time of recurrence [8, 10]. The presence of ascitic liquid is due to transcoelomic dissemination, which is the most common mode of metastasis in HGSOC [8, 11, 12], and is indicative of poor prognosis [13, 14].

Transcoelomic dissemination consists of a multistep process that starts with the shedding of cancer cells from the primary tumor. As HGSOC cells spread to the abdominal space, they promote the production of ascites [11]. During their flotation, malignant cells will require an escape from anoikis, an anchorage‐independent cell death [15]. For this reason, shed cells from the primary tumor aggregate as multicellular clusters. This clustering represents an essential survival mechanism for transcoelomic dissemination [16]. These aggregates, known as tumorspheres, have been described to possess a cancer‐associated fibroblast (CAF) backbone, which provides recruited ovarian cancer cells with indispensable signals for the cluster's sustainment [17]. Moreover, tumorspheres represent the invasive and chemoresistant cellular population fundamental to ovarian cancer metastatic spread [18]. They are also the result of an adaptive process in order to survive immunosurveillance within the abdominal cavity [9] and exhibit pronounced capacity to adhere to mesothelium and penetrate the extracellular matrix (ECM) [11, 15]. Furthermore, it has been observed that tumorspheres also arise from collective detachment [19] and that they provide an evolutionary advantage in tumor progression over single cells [3]. However, the precise molecular mechanisms governing ovarian cancer tumorsphere formation and survival within the ascites microenvironment remain poorly understood.

In this study, our group sought to decipher the key signals that drive transcoelomic dissemination to identify new vulnerabilities specific to malignant tumorspheres. Based on tumorspheres isolated from ascitic liquid samples from HGSOC patients and on ovarian cancer spheroid *in vitro* and *in vivo* models, we identified the platelet‐derived growth factor receptor beta (PDGFRβ)–fibronectin axis as a key mechanism for ovarian tumorsphere formation and metastatic spread.

## 2 Materials and methods

### 2.1 HGSOC patients' samples

Tissue sections for immunohistochemistry assays were provided by the tumor tissue bank of the Bellvitge University Hospital (HUB)‐IDIBELL, and ascitic liquid samples were provided by the Gynecology Service of HUB (January 2012 to January 2023). All samples were processed following standard operating procedures, with the appropriate approval of the Clinical Investigation Ethics Committee of HUB (meeting minutes 18/11, ref. PR236/11) and conformed to the principles set out in the WMA Declaration of Helsinki. A signed informed consent was obtained from each patient. Clinical characteristics of each patient are summarized in Fig. S1A and Table S1.

### 2.2 AS‐OV cells and tumorspheres isolation and culture

Ascitic liquid extracted from HGSOC patients was split into 50 mL tubes and centrifuged at 500 *g* for 8 min. Supernatants were discarded and pellets were lysed with 1× PBS‐diluted RBC Lysis Buffer (Cat. Num. #420301; BioLegend, San Diego, CA, USA) and centrifuged again at 500 *g* for 8 min. Afterwards, pellets were washed with PBS and then incubated with previously prepared magnetic beads conjugated with EpCAM antibody (Dynabeads™ M‐280 Sheep IgG anti‐rabbit, Cat. Num. #11203D; Thermo Fisher Scientific, Waltham, MA, USA; EpCAM antibody produced in rabbit, Cat. Num. #HPA026761; Sigma‐Aldrich, Saint Louis, MO, USA), in 0.5% BSA PBS solution for 1 h at room temperature (RT). Finally, isolated ascites EpCAM+ (AS‐OV) cells or tumorspheres were seeded in 199 Earle's Salts cell culture medium (Cat. Num. #11554426; Gibco, Waltham, MA, USA) and MCDB105 cell culture medium (Cat. Num. #117‐500; Cell Applications, San Diego, CA, USA) 1 : 1 supplemented with 10% FBS, 2 mm l‐glutamine, 1 mm pyruvate and antibiotics (10^4^ units·mL^−1^ and 10^4^ mg·mL^−1^ of Penicillin and Streptomycin, respectively) in cell culture plates; or 3D conditions, respectively, and subjected to the corresponding treatments. Regarding 2D culture, AS‐OV cells were maintained through 2–3 passages until cells became senescent and hence were not considered for further assays. As for AS‐OV tumorspheres, 3D cultures were maintained through 3–4 splits, until tumorspheres stopped proliferating and exhibited a necrotic center with dead cells, or even started losing their structure. When this was observed, they were no longer considered for further assays. AS‐OV tumorsphere culture splits involved dividing the volume contained in each plate into some new plates, adding new, fresh 3D conditions medium up to the desired final volume. Further details about 2D cell passaging and 3D culture conditions are in Section 2.4.

### 2.3 Animal procedures

For the generation of orthotopic tumors, immunocompromised female mice 7–8 weeks old (Athymic Nude‐Foxn1^nu^; Envigo, Horst, The Netherlands) were anesthetized with isoflurane and 1 million SKOV3 cells were injected into the ovary in a 20 μL Dulbecco's modified Eagle medium (DMEM) solution. After 1 month, animals were euthanized and a necropsy was performed so as to resect primary tumors and metastases for further RNA extraction studies. Regarding the study of omental metastases after doxycycline‐induced PDGFRβ silencing, 500 000 SKOV3 dox‐sh‐PDGFRB cells previously treated with 1 μg·mL^−1^ doxycycline or vehicle, and resuspended in 1 mL FBS‐free DMEM medium, were intraperitoneally injected into immunocompromised female mice 7–8 weeks old (Athymic Nude‐Foxn1^nu^; Envigo). Animals were administered with 400 mg doxycycline and 6 g glucose per 200 mL of drinking water. After 10 days, luciferase activity was assessed by use of an IVIS® Spectrum after intraperitoneal injection of 200 μL 15 μg·mL^−1^ Luciferin. Animals were euthanized and a necropsy was performed in order to assess omental disseminations. Regarding spheroid intraperitoneal implantation experiments, spheroids were generated from the corresponding cell lines (sh‐CTRL‐Luc and sh‐PDGFRB‐Luc) as described below in Section 2.5. Two hundred and fifty spheroids were resuspended in 500 μL of FBS‐free DMEM/F12 and intraperitoneally injected into NSG female mice 7–8 weeks old (NOD‐scid IL2Rgamma null, IDIBELL animal facility). Luciferase activity was assessed by use of an IVIS® Spectrum after intraperitoneal injection of 200 μL 15 μg·mL^−1^ Luciferin, once a day throughout the following week, and once a week until reaching 8 weeks. After this period, animals were euthanized and a necropsy was performed so as to assay luciferase activity of disseminations and metastases. Images were processed with living image software (Spectral Instruments Imaging, Tucson, AZ, USA) and ROIs were quantified. Sunitinib (SU11248), cisplatin, and combined treatment were initiated 2 weeks after spheroid intraperitoneal implantation. During these 2 weeks, luciferase activity was assessed twice a week in order to monitor disseminations' location and growth. Then, mice presenting similar ROI median radiances were uniformly distributed across the three treatment groups so that median radiances per treatment group were equivalent. Sunitinib was administered five times a week by oral gavage (40 mg·kg^−1^ body weight; Cat. Num. #HY‐10255A; MedChemExpress, Monmouth Junction, NJ, USA) during 4 weeks, and drug administration was accompanied by subcutaneous injection of 500 μL of 5% serum glucose. Cisplatin was administered once a week by intraperitoneal injection (4 mg·kg^−1^ body weight; Cat. Num. #CRN008NJH; Accord Healthcare, Durham, NC, USA) only for the first 2 weeks. Luciferase activity was assessed once a week throughout the following month. After this period, animals were euthanized and a necropsy was performed so as to assay luciferase activity of disseminations and metastases. None of administered treatments nor mice manipulation had a significant effect on mice body weight and behavior. As an immunocompromised animal, mice were located in specific pathogen‐free area in our animal facility where health, activity, environment, housing, food, and drink were strictly monitored. All studies were approved by the local committee for animal care (IDIBELL and Generalitat de Catalunya, DTES 9731).

### 2.4 Cell culture

SKOV3 cells (SK‐OV‐3, RRID: CVCL_0532; Sigma‐Aldrich) and SKOV3‐derived cell clones were grown in DMEM (Cat. Num. BE12‐614Q; Lonza, Morristown, NJ, USA) supplemented with 10% FBS, 2 mm l‐glutamine, 1 mm pyruvate, and antibiotics (10^4^ units·mL^−1^ and 10^4^mg·mL^−1^ of Penicillin and Streptomycin, respectively). A2780 cells (RRID: CVCL_0134, kindly donated by Giménez‐Bonafé from Universitat de Barcelona, Barcelona, Spain) were grown in RPMI 1640 Medium supplemented with 10% FBS, 2 mm l‐glutamine, 1 mm pyruvate and antibiotics (10^4^ units·mL^−1^ and 10^4^ mg·mL^−1^ of Penicillin and Streptomycin, respectively). All cell lines have been authenticated in the past 3 years by IDEXX BioAnalytics using STR‐based DNA profiling and multiplex PCR. Human CAFs isolated from colorectal cancer liver metastases (CRC‐LM; CAF1‐4, kindly donated by Garcia‐Molleví from ProCURE Program, ICO‐IDIBELL, L'Hospitalet de Llobregat, Spain) were cultured in DMEM/F12 supplemented with 10% FBS, 2 mm l‐glutamine, 1 mm pyruvate, and antibiotics. All cell lines used in this study were cultured at 5% CO_2_ and 37 °C and tested negative for mycoplasma. They were maintained through up to 20 cell passages. Cell passaging involved washing cells with 5 mL PBS 1× and then subjecting them to trypsin (Cat. Num. #15090046; Gibco) for 10 min at 37 °C, which was subsequently inactivated with new, fresh cell culture medium. Regarding suspension cultures for anoikis resistance assays, 200 000 cells were seeded in each well of 6‐well suspension plates (Cat. Num. #833920500; Sarstedt, Newton, NC, USA) in 3D conditions, that is, DMEM/F12 freshly supplemented with 1× B27 (Cat. Num. #17504044; Gibco), 10 ng·mL^−1^ human recombinant bFGF (Cat. Num. #F0291; Sigma‐Aldrich), and 0.02 ng·mL^−1^ EGF (Cat. Num. #E9644; Sigma‐Aldrich). As for spheroid formation assays, 400 000 cells were seeded in nontreated plates (Cat. Num. #38070; STEMCELL Technologies, Vancouver, BC, Canada) in 3D conditions and upon the corresponding treatments. For chimeric spheroid generation, 200 000 sh‐PDGFRB cells prestained with CellTrace™ Violet dye (Cat. Num #C34571; Thermo Fisher Scientific) and 200 000 CAFs prestained with CellTrace™ CFSE dye (Cat. Num. #C34570; Thermo Fisher Scientific) were seeded using the same specified procedure for 3D conditions culture. After 3 days at 37 °C and 5%CO_2_, three images of randomly picked fields were obtained by use of a Leica DM IL LED Tissue Culture Microscope (Leica Microsystems, Allendale, NJ, USA) at 10× and the number and diameter of spheroids contained within an area of interest (AOI) of 100 000 μm^2^ or the entire field were quantified by imagej/fiji software [20]. Factors and drugs used for cell, spheroid, and tumorsphere treatment are listed in Table S2.

### 2.5 Generation of lentiviral vectors and cell transduction

For the generation of stable, human PDGFRβ‐silencing cell clones (sh‐CTRL and sh‐PDGFRB), 293FT cells were transfected with MISSION® pLKO.1‐puro Empty Vector Control Plasmid (Cat. Num. #SHC001; Sigma‐Aldrich) and anti‐PDGFRβ shRNA MISSION® pLKO.1 lentiviral vectors, along with the envelope expressing pMD2.G (Cat. Num. #12259; Addgene, Watertown, MA, USA) and packaging psPAX2 (Cat. Num. #12260; Addgene) lentiviral plasmids, and incubated for 48 h at 37 °C and 5% CO_2_. Afterward, 200 000 SKOV3 cells were seeded in 6‐well plates along with 293FT cells' supernatants and 8 μg·mL^−1^ Polybrene and subjected to a 600 *g* spin for 90 min at 32 °C. Then, cells were incubated for 24 h at 37 °C and 5% CO_2_ and the medium was changed the day after. Two days later, antibiotic selection with 10 μg·mL^−1^ Puromycin (Cat. Num. #12122530; Gibco) was performed and resistant cell clones were selected. For the generation of doxycycline‐inducible, human PDGFRβ‐silencing cell clones (dox‐sh‐CTRL, dox‐sh‐PDGFRB1, and dox‐sh‐PDGFRB2), the same protocol was followed, transfecting 293FT cells in this case with the TRIPZ Inducible Lentiviral Human PDGFRB shRNA from Dharmacon Reagents (Lafayette, CO, USA). For the generation of Luciferase‐expressing cells (sh‐CTRL‐Luc and sh‐PDGFRB‐Luc), the same protocol described above was followed, transfecting 293FT cells in this case with the pLentipuro3/TO/V5‐GW/EGFP‐firefly luciferase plasmid (Cat. Num. #119816; Addgene). Spheroids were generated from all cell lines and subjected to a luciferase activity assay (described below) to ensure that all of them presented the same basal luciferase activity.

### 2.6 Luciferase activity assay

Spheroids from SKOV3 sh‐CTRL‐Luc and sh‐PDGFRB‐Luc cell lines were generated, and the volume corresponding to 250 spheroids was collected for the assay. Such volume was centrifuged at 600 *g* for 5 min at 4 °C, and then pellets were washed in 500 μL of cold PBS and centrifuged again at 600 *g* for 5 min at 4 °C. Pellets were then lysed with 100 μL of Reporter Lysis 5× Buffer (Cat. Num. #E397A; Promega, Madison, WI, USA) and subjected to protein quantification by use of Pierce™ BCA Protein Assay Kit (Cat. Num. #23225; Thermo Fisher Scientific). In parallel, 40 μL of Luciferase Assay Reagent (Cat. Num. #E1483; Promega) was placed in each well to be assayed of a 96‐well assay white plate (Cat. Num. #3610; Corning Inc, New York, NY, USA) along with 2 μL of lysate, and light was read at 560 nm in a VICTOR X Multilabel Plate Reader (Perkin Elmer, Waltham, MA, USA). Finally, obtained measures were corrected by amount of protein per sample.

### 2.7 Cell viability assays

SKOV3, A2780, and AS‐OV cells were counted by aid of a Neubauer Chamber, and 5000 cells were seeded per well in a 96‐well plate in supplemented DMEM cell culture medium for SKOV3 cells, supplemented RPMI 1640 cell culture medium for A2780 cells and supplemented 199:MCDB105 medium for AS‐OV cells. The day after, the corresponding treatments were added and cells were incubated at 37 °C 5%CO_2_ for 3 days. To proceed with the revealing, cells were fixed with methanol and stained with 0.1% Cristal Violet (Cat. Num. #C6158; Sigma‐Aldrich) solution. Finally, each well was resuspended with 10% methanol and 5% acetic acid solution, and absorbance was read at 595 nm by use of a spectrophotometer.

### 2.8 RNA extraction, reverse transcription, and real‐time PCR

SKOV3 and AS‐OV cells were washed thrice with PBS and harvested with 500 μL Trizol Reagent (Cat. Num. #15596018; Ambion, Thermo Fisher Scientific) and then to a final volume of 1 mL. Regarding spheroid and ascitic liquid samples, all the medium was collected and centrifuged at 600 *g* for 5 min at 4 °C, and then pellets were washed in 500 μL of cold PBS and centrifuged again at 600 *g* for 5 min at 4 °C. Afterward, pellets were resuspended in 500 μL Trizol Reagent and then to a final volume of 1 mL. After that, 200 μL of chloroform was added per mL of Trizol, and samples were vortexed for 2 min and centrifuged at 12 000 *g* for 15 min at 4 °C, in order to obtain three phases. The upper one was collected and mixed 1 : 1 with isopropanol. Samples were stored for 24 h at −20 °C and afterward centrifuged at 12 000 *g* for 30 min at 4 °C. Then, supernatants were decanted and 1 mL of 70% ethanol was added. Samples were centrifuged again at 12 000 *g* for 5 min at 4 °C and decanted. Then, pellets were resuspended in RNAse‐free water with 10× Turbo DNAse Buffer and DNase I (Cat. Num. AM1907; Thermo Fisher Scientific) to a final volume of 30 μL and incubated for 25 min at 4 °C. Finally, 3 μL of DNAse Inactivation Reagent was added. After being extracted, RNA was quantified in a NanoDrop™ spectrophotometer (Thermo Fisher Scientific) and nucleic acid purity was further determined by the obtained 260/280 and 260/230 ratios. Then, 2000 ng of RNA was added to a final volume of 20 μL and incubated for 10 min at 65 °C. Afterward, cDNA was obtained by reverse transcription reaction by use of a Reverse Transcription Kit (Cat. Num. #10400745; Applied Biosystems, Thermo Fisher Scientific), programmed to be performed in the GeneAmp™ PCR System 9700 Thermal Cycler (Thermo Fisher Scientific) at 37 °C during 2 h. After that, RT‐PCR was performed in a 384‐well plate and with SYBR™ Green Master Mix (Cat. Num. A25742; Applied Biosystems, Thermo Fisher Scientific). RT‐PCR was programmed to be performed up to 40 cycles of amplification in the LightCycler® 480 System (Roche Molecular Biochemicals, Basel, Switzerland). β‐Actin was used as a housekeeping gene, and CP values were calculated according to the formula: $10\;000\times 2^{-\Delta C_{t}}$. Primers used are listed in Table S3.

### 2.9 Western blot

SKOV3 cells were washed thrice in cold PBS and harvested with 4 °C RIPA lysis buffer (PBS pH 7.4, SDS 0.1%, NP‐40 1%, sodium deoxycholate 0.5%) supplemented with protease and phosphatase inhibitors (100 μm PMSF, 1 μm pepstatin A, 1 μg·mL^−1^ leupeptin, 4 μg·mL^−1^ aprotinin, 0.1 μg·mL^−1^ benzamidine, 200 μm sodium orthovanadate, 10 mm NaF, 40 mm β glycerol phosphate, and 5 mm EDTA). Regarding spheroid and tumorsphere samples, all the medium was collected and centrifuged at 600 *g* for 5 min at 4 °C, and then pellets were washed in 500 μL of cold PBS and centrifuged again at 600 *g* for 5 min at 4 °C. Afterward, pellets were lysed with RIPA lysis buffer supplemented with protease and phosphatase inhibitors. Lysed samples were subjected to centrifugation at 16 000 g for 15 min at 4 °C, and supernatants were collected. Samples' protein concentration was quantified by use of Pierce™ BCA Protein Assay Kit (Cat. Num. #23225; Thermo Fisher Scientific) and the same amount of protein in all samples was then separated by SDS/PAGE, using the Laemmli buffer system. Separated proteins were then transferred to Immobilon‐P membranes (Cat. Num. #IPVH00010; Millipore, Burlington, MA, USA) in 25 mm Tris/HCl, 190 mm glycine, and 10% methanol at 4 °C, which were then blocked in 5% fat‐free powdered milk TBS (10 mm Tris/HCl, 150 mm NaCl, pH 7.4) for 1 h at RT and blotted overnight with the corresponding primary antibodies in 1% fat‐free powdered milk TBS. Membranes were washed thrice for 10 min with 0.1% Triton X‐100 TBS and incubated with anti‐rabbit Ig (Cat. Num. #NA934; Amersham Pharmacia Biotech, Little Chalfont, UK) or anti‐mouse Ig (Cat. Num. #NXA931; Amersham Pharmacia Biotech) horseradish peroxidase‐linked antibodies, in 1% fat‐free powdered milk TBS. Membranes were washed again with 0.1% Triton X‐100 TBS and incubated for 1 min in ECL solution (obtained by mixing 1 m Tris pH 8.5, 90 mm P‐coumaric acid, 250 mm Luminol in DMSO; and 30% hydrogen peroxide in a 1 mL to 3 μL proportion). ECL signal was detected with the ChemiDoc™ MP imaging system, and volumetric analysis was performed using imagelab from Bio‐Rad (Hercules, CA, USA). Primary antibodies used for western blot are listed in Table S4.

### 2.10 Immunofluorescence assays

Spheroids or tumorspheres were collected and centrifuged at 600 *g* for 5 min and then washed in 500 μL PBS. Afterward, they were fixed with 4% paraformaldehyde (PFA) during 2 h in gentle balancing. Then, samples were washed twice during 10 min with 0.1% Triton X‐100 0.1% Azide PBS and permeabilized in 1% Saponin PBS for 45 min in gentle balancing. After, spheroids or tumorspheres were incubated overnight with 3% BSA 0.1% Azide in PBS blocking solution at 4 °C in agitation. Afterward, they were incubated for 72 h in 0.5% BSA 0.1% PBS 1 : 100 Ki‐67 (Cat. Num. MA5‐14520; Invitrogen), fibronectin (Cat. Num. #ab2413; Abcam, Cambridge, UK) or EpCAM (Cat. Num. HPA026761; Sigma‐Aldrich) antibody solution, at 4 °C in agitation. Then, spheroids or tumorspheres were washed twice during 10 min with 0.1% Triton X‐100 0.1% Azide PBS and incubated for 3 h in 0.5% BSA 0.1% PBS 1 : 200 Alexa Fluor 488 goat anti‐Rabbit (Cat. Num. #A‐11008; Invitrogen) or Alexa Fluor 546 goat anti‐Rabbit (Cat. Num. #A‐11010) antibody solution, covered from light, in gentle balancing. Afterward, samples were washed twice during 10 min with 0.1% Triton X‐100 0.1% Azide PBS. Afterward, spheroids or tumorspheres were incubated in 1 μg·mL^−1^ 4′,6‐diamidino‐2‐phenylindole (DAPI) in PBS for 1 h, washed once during 10 min with 0.1% Triton X‐100 0.1% Azide PBS, and mounted in Fluoromont™ Aqueous Mounting Medium (Cat. Num. F‐4680; Sigma‐Aldrich) on glass slides. Fluorescence was assessed under 40× magnification on a Leica SP‐5 confocal microscope (Leica Microsystems), and images of 10–15 randomly picked spheroids per condition were obtained. Quantification of Ki67‐positive nuclei was automatically performed *in silico* by the use of imagej software's (NIH, Bethesda, MD, USA) StarDist, Bio‐Formats and CSBDeep plugins.

### 2.11 Immunohistochemistry assays

Paraffin‐embedded tissue sections were deparaffinized in xylene and rehydrated in downgraded alcohols and distilled water. Antigens were retrieved under high‐pressure conditions for 4 min in citrate buffer at pH 6 (for c‐Kit, PDGFRβ and EpCAM) or pH 9 (for PDGFRα), and endogenous peroxidases were deactivated. Samples were blocked with TBS 1× 0.5% Triton 6% donkey serum solution before primary antibody incubation overnight at 4 °C. Rabbit polyclonal anti‐c‐Kit ready to use (Cat. Num. #A4502; Dako, Agilent Technologies, Santa Clara, CA, USA), rabbit polyclonal IgG anti‐PDGFRβ (Cat. Num. #sc339 P‐20; Santa Cruz Biotechnology, Santa Cruz, CA, USA) at 1 : 150, rabbit polyclonal anti‐EpCAM (Cat. Num. HPA026761; Sigma‐Aldrich) at 1 : 70, and rabbit polyclonal IgG anti‐PDGFRα (Cat. Num. #5241; Cell Signaling Technology, Danvers, MA, USA) at 1 : 70 were used as primary antibodies. Sections were then incubated for 30 min at RT with specific secondary anti‐rabbit IgG EnVision Dual Link antibody (Cat. Num. #K4065; Agilent Technologies, Santa Clara, CA, USA), followed by the Dako DAB+ Substrate Chromogen developing system (Cat. Num. #GV825; Dako, Agilent Technologies). Samples were counterstained with hematoxylin and visualized under light microscopy. Protein stainings were measured as a grading scale, defined as follows: no detectable signal (0 points), low‐intensity signal (1 point), moderate‐intensity signal (2 points), or high‐intensity signal (3 points). Labeling frequency was scored as the percentage of positive tumor cells. The multiplicative index of intensity and labeling frequency was used in our analysis, as we had previously described elsewhere [21, 22].

### 2.12 Bioinformatics analyses

Expression data from microarray and RNAseq experiments provided by two independent studies [23, 24] publicly available in Gene Expression Omnibus (GEO accession numbers GSE14407 and GSE137237, respectively) were downloaded and normalized to LOG2 scale. Obtaining of data and posterior ordering and graphical representation were performed by r programming in the rstudio programming environment (v. 2022.12.0+353), using reshape2 (v. 1.4.4), ggplot2 (v. 3.4.0), ggsignif (v. 0.6.4), ggpubr (v. 0.5.0), and dplyr (v. 1.0.10) packages. Significant differences were assessed using Mann–Whitney *U*‐test when comparing between two groups and considered when *P* < 0.05 (*<0.05). For correlation studies, gene signatures (Table S5) were downloaded from the Molecular Signature Database (MSigDB) [25, 26]. Signature scores were computed using the single‐sample gene set expression analysis (ssGSEA) algorithm calculated within the gene set variation analysis (gsva) package (v. 1.46.0) [27]. Pearson's correlation was used for calculating *P* and *R* values.

### 2.13 Statistical analyses

Statistical analyses were performed with graphpad prism (GraphPad Software Inc, San Diego, CA, USA, versions 6.01 and 8.3). Unless otherwise specified, data are presented Mean ± SD, and each graph dot represents an independent experiment in case of cells and spheroids, and an independent patient sample in case of experiments performed with AS‐OV cells and tumorspheres. Significant differences were assessed using Mann–Whitney *U*‐test when comparing between two groups, and significant differences were considered when *P* < 0.05 (***<0.001, **<0.01, *<0.05).

## 3 Results

### 3.1 Sunitinib treatment hampers ovarian cancer tumorspheres formation

**Fig. 1:**
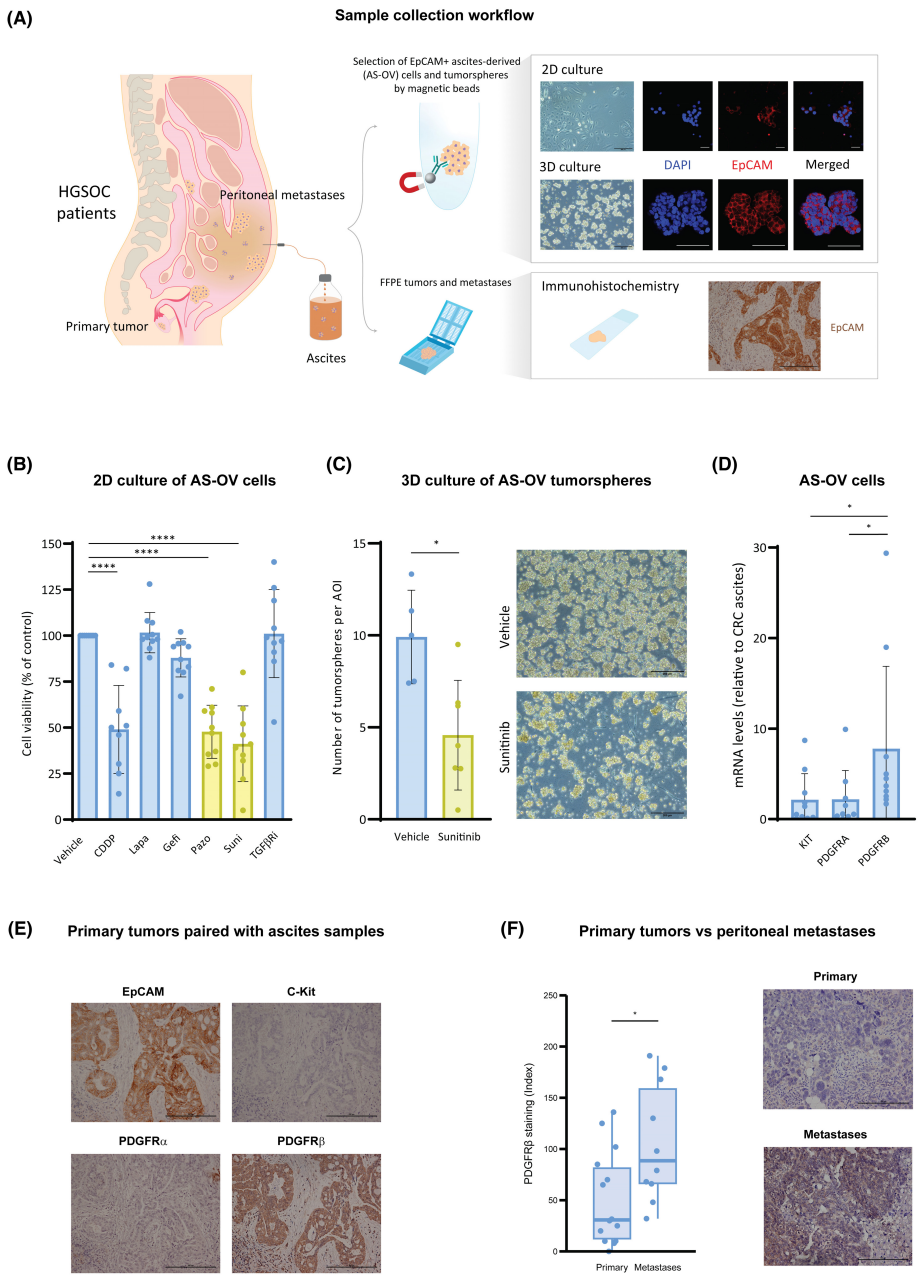
Sunitinib blocks tumorspheres formation and the disseminative capacity of ovarian tumor cells during metastatic spread. (A) Sample collection workflow. (B) Cell viability of EpCAM+ ascites‐derived (AS‐OV) cells cultured in 2D upon cisplatin (CCDP), lapatinib (Lapa), gefitinib (Gefi), pazopanib (Pazo), sunitinib (Suni), and TGFβR inhibitor (TGFβRi) treatments. Vehicle is DMSO. (C) Number of AS‐OV tumorspheres per area of interest (AOI) upon sunitinib treatment. (D) mRNA expression by real‐time PCR of sunitinib targets in AS‐OV cells relative to colorectal cancer (CRC) ascites‐derived cells. (E) Protein expression of sunitinib targets in primary tumors paired with ascites samples. (F) Protein expression of PDGFRβ in paired primary tumors and metastases. Representative images of tumorspheres in C (out of *n* = 7 samples and 3 images per sample) and tissue sections in E (out of *n* = 14 samples and 3 images per sample) and F (out of *n* = 14 primary tumor and *n* = 10 peritoneal metastases; and 3 images per sample) are shown. Scale bars: 200 μm. Data are presented mean ± SD, and each graph dot represents an independent patient sample in B (*n* = 10), C (*n* = 12), D (*n* = 10) and F (*n* = 24). Significant differences were assessed using Mann–Whitney *U*‐test when comparing between two groups, and considered when *P* < 0.05 (****<0.0001, *<0.05).

In order to assess the specific impact of cancer treatments on disseminating tumor cells, we studied ascites samples as well as their paired Formalin‐Fixed Paraffin‐Embedded (FFPE) primary tumors and peritoneal metastatic lesions (Fig. 1A) from a cohort of 32 treatment‐naïve HGSOC patients that had undergone diagnostic laparoscopy or primary debulking surgery (Fig. S1A and Table S1). Tumor cells (AS‐OV), either as single cells or tumorspheres, were isolated from fresh ascites using EpCAM^+^ selection and cultured in 2D or 3D conditions for subsequent experiments. First, AS‐OV cells were subjected to a multidrug screening assay, comparing cisplatin, the standard of care at the time of the study, with a series of anticancerous treatments [28, 29, 30, 31, 32]. Among these drugs, only sunitinib and pazopanib, multireceptor tyrosine kinase inhibitors of the same family (RTKI) [33], exhibited a comparable effect to cisplatin reducing AS‐OV cell viability by more than 50% (Fig. 1B). Tumorspheres are critical for dissemination and metastasis formation; therefore, we aimed to evaluate the effect of sunitinib in 3D conditions. When tumorspheres isolated from ascitic liquid samples were cultured in 3D conditions and subjected to sunitinib treatment, the number of tumorspheres was significantly impaired (Fig. 1C). These observations suggested that one or some of sunitinib targets could be involved in the formation of ovarian cancer tumorspheres. Consequently, we evaluated their mRNA expression by real‐time PCR and discovered PDGFRβ mRNA levels to be the most elevated in AS‐OV cells and significantly increased in ovarian cancer ascites compared to those of nonovarian cancerous ascites samples, such as colorectal cancer (CRC)‐derived ascites (Fig. 1D). The increased expression of PDGFRβ was confirmed at the protein level in EpCAM+ cells from those primary tumors paired with ascites samples analyzed, while c‐Kit and PDGFRα were almost indetectable (Fig. 1E and Fig. S1B). Notably, the expression of PDGFRβ was higher in peritoneal lesions compared with primary tumors, indicating an enhanced metastatic potential of PDGFRβ‐expressing cells (Fig. 1F). These results were validated by *in silico* analyses of HGSOC patients data from two independent datasets [23, 24], where PDGFRβ mRNA expression was found to be higher in HGSOC patients' metastases in comparison with primary tumors (Fig. S1C) and also in primary tumors from advanced HGSOC (FIGO III‐IV) in comparison with those classified in early stages (FIGO Ic‐IIb; Fig. S1D). Altogether, our results indicate that PDGFRβ could play a relevant role in the dissemination capacity of ovarian tumor cells.

**Fig. 2:**
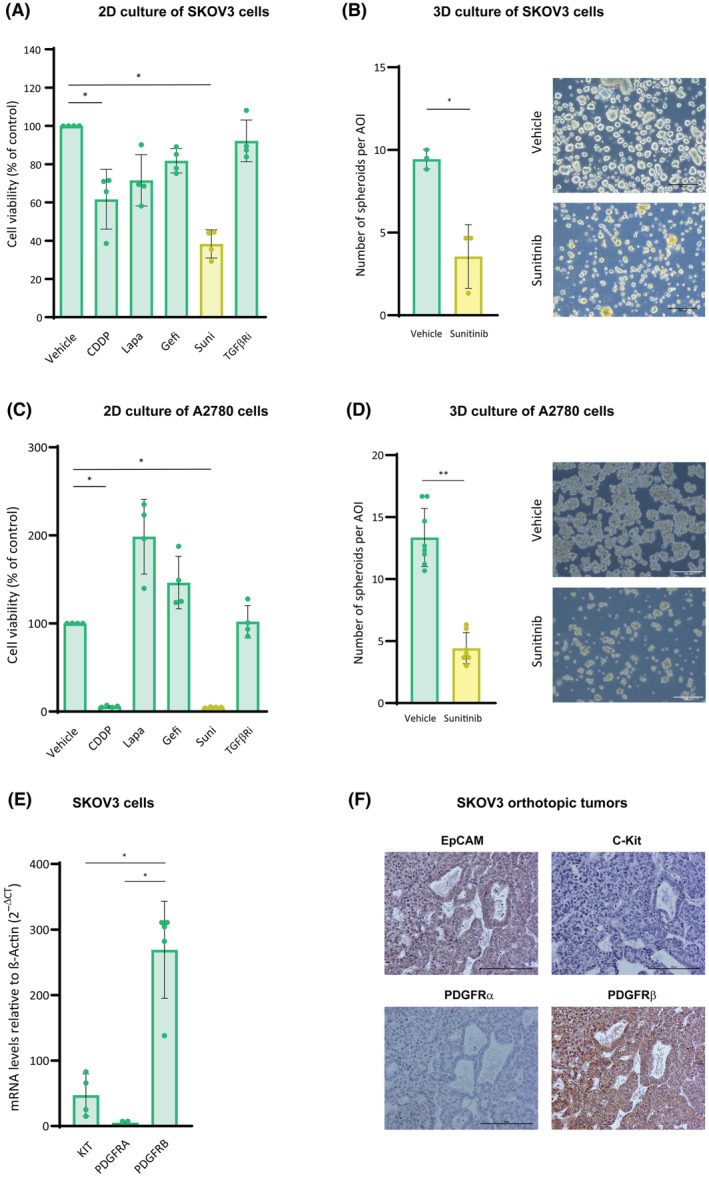
Sunitinib blocks spheroid formation in ovarian cancer cells. (A, C) Cell viability of SKOV3 (A) and A2780 (C) cells cultured in 2D upon cisplatin (CCDP), lapatinib (Lapa), gefitinib (Gefi), sunitinib (Suni), and TGFβR inhibitor (TGFβRi) treatments. Vehicle is DMSO. (B, D) Number of spheroids per area of interest (AOI) of SKOV3 (B) and A2780 (D), upon sunitinib treatment. (E) mRNA expression of sunitinib targets in SKOV3 cells. (F) Protein expression of sunitinib targets in SKOV3 orthotopic tumors. Representative images of spheroids in B and D (out of *n* = 3 and *n* = 7 independent experiments, respectively, and 3 images per experimental condition) and tissue sections in F (out of *n* = 3 samples, and 3 images per sample) are shown. Scale bars: 200 μm. Data are presented mean ± SD, and each graph dot represents an independent experiment in A (*n* = 4), B (*n* = 3), C (*n* = 4), D (*n* = 7) and E (*n* = 4). Significant differences were assessed using Mann–Whitney *U*‐test when comparing between two groups and considered when *P* < 0.05 (**<0.01, *<0.05).

To further validate our results in cellular models, ascites‐derived SKOV3 human ovarian cancer cells were subjected to the multidrug screening mentioned above. Sunitinib exhibited the greatest effect on SKOV3 cell survival (Fig. 2A), and when SKOV3 cells were seeded in 3D conditions and subjected to sunitinib treatment, it significantly decreased spheroid formation capacity (Fig. 2B). Similar results were obtained in different HGSOC cancer cell line, A2780 (Fig. 2C,D). Moreover, the expression of *PDGFRB* was the highest among all PDGF receptors in SKOV3 cells (Fig. 2E), and protein expression of PDGFRβ was also prominent in orthotopic tumors generated in immunodeficient female mice compared with c‐Kit and PDGFRα (Fig. 2F). Collectively, all these observations suggested that PDGFRβ is necessary for ovarian cancer cell aggregation and tumorsphere formation, which was further validated in four different ovarian cancer 3D models.

### 3.2 Ovarian cancer cells depend on PDGFRβ for tumorsphere formation and *in vivo* dissemination

**Fig. 3:**
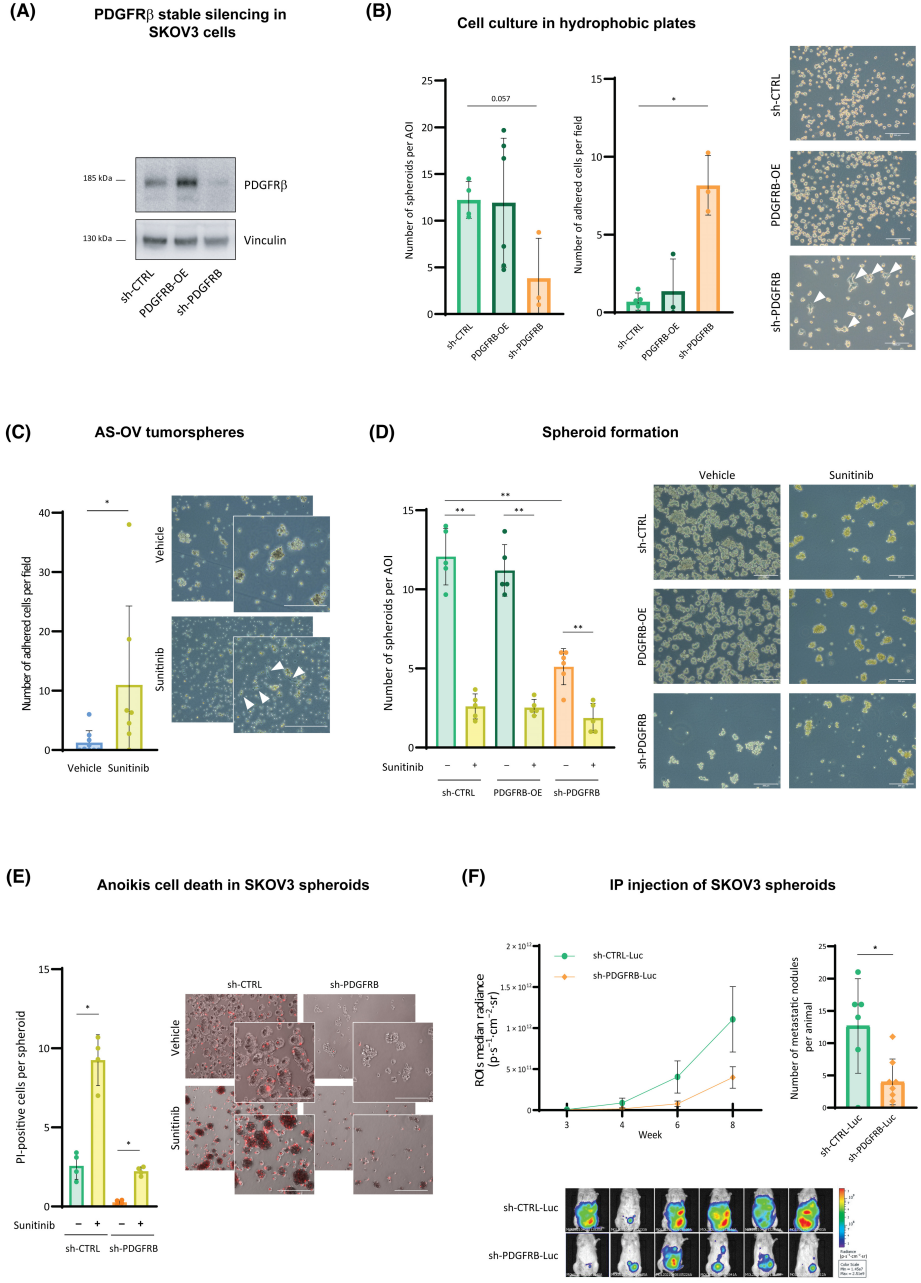
PDGFRβ plays a role in ovarian cancer tumorsphere and spheroid formation and in the disseminative capacity of ovarian tumor cells during metastatic spread. (A) Western blot for PDGFRβ expression in sh‐CTRL, PDGFRB‐OE, and sh‐PDGFRB cells. Vinculin was used as loading control. A representative image of three independent experiments is shown. (B) Number of spheroids (left panel) and number of adhered cells per field (middle panel) of sh‐CTRL, PDGFRB‐OE, and sh‐PDGFRB cells cultured in hydrophobic cell culture plates. Right panel: representative images of spheroids and adhered cells (white arrowheads). (C) Number of adhered AS‐OV cells (white arrowheads) per field upon sunitinib treatment of AS‐OV tumorspheres. (D) Number of spheroids of sh‐CTRL, PDGFRB‐OE, and sh‐PDGFRB cells cultured in 3D conditions in nontreated plates, upon sunitinib treatment. (E) Anoikis cell death by PI staining of sh‐CTRL and sh‐PDGFRB spheroids upon sunitinib treatment. (F) Left, upper panel: ROIs radiance (expressed in p·s^−1^·cm^−2^·sr^−1^) of immunodeficient female mice intraperitoneally injected with 250 spheroids generated from sh‐CTRL‐Luc (*n* = 6 mice) and sh‐PDGFRB‐Luc (*n* = 8 mice) cells. Data in this graph are shown mean ± SEM. Right, upper panel: metastatic nodules per animal. Lower panel: representative images taken with the IVIS® Spectrum. Colorized areas indicate presence and size of disseminations. Data presented are shown Mean ± SD unless otherwise specified and each graph dot represents an independent experiment in B (*n* = 4), D (*n* = 5) and E (*n* = 4); an independent patient sample in C (*n* = 6 per group), and an independent animal in F (*n* = 6 mice per group). Representative images are shown in B (out of *n* = 4 independent experiments, and 3 images per experimental condition), C (out of *n* = 6 samples per group, and 3 images per sample), D (out of *n* = 5 independent experiments, and 3 images per experimental condition) and E (out of *n* = 4 independent experiments, and 3 images per experimental condition), and scale bars are of 200 μm. Significant differences were assessed using Mann–Whitney *U*‐test when comparing between two groups, and considered when *P* < 0.05 (**<0.01, *<0.05).

To confirm the role of PDGFRβ in the process of ovarian cancer transcoelomic dissemination, doxycycline‐inducible, PDGFRβ‐silencing SKOV3 cell clones were generated (Fig. S2A). When cultured in hydrophobic cell culture plates so as to assay their anoikis resistance, upon doxycycline, dox‐sh‐PDGFRB cells exhibited reduced growth in suspension conditions and adhered to the plastic instead (Fig. S2B). Moreover, the number of omental metastases generated after orthotopic implantation of these cells was lower under doxycycline induction (Fig. S2C). These observations suggested that ovarian cancer cells rely on PDGFRβ to survive in suspension conditions and disseminate *in vivo*. To validate these results, a SKOV3 cell model for stable silencing of PDGFRβ (sh‐PDGFRB) was generated (Fig. 3A). When cultured in hydrophobic cell culture plates, cells with basal (sh‐CTRL) or higher levels of PDGFRβ (PDGFRB‐OE) were able to grow in suspension (Fig. 3B, left panel), whereas sh‐PDGFRB cells were not able to do it and mostly adhered to the plastic (Fig. 3B, middle and right panels). Interestingly, this behavior was also observed in AS‐OV cells cultured in 3D conditions upon sunitinib treatment (Fig. 3C). In addition, when cultured in 3D conditions, sh‐PDGFRB cells exhibited reduced spheroid formation capacity in comparison with sh‐CTRL and PDGFRB‐OE cells (Fig. 3D) and presented mostly as multicellular aggregates rather than compact spheroids. sh‐CTRL and PDGFRB‐OE cells were not able to generate spheroids either when subjected to sunitinib treatment, recapitulating the phenotype observed in sh‐PDGFRB (Fig. 3D). Sunitinib did still exert a significant effect on sh‐PDGFRB aggregates, which was due to an increase in anoikis cell death (Fig. 3E). Such increase in cell death due to sunitinib treatment was also observed in other ovarian cancer cell lines (Fig. S2D). Overall, all these data indicated that when PDGFRβ is silenced or pharmacologically inhibited, ovarian cancer cells lose their capacity to aggregate in suspension and die. To confirm this, 250 luciferase‐expressing sh‐CTRL (sh‐CTRL‐Luc) or sh‐PDGFRB (sh‐PDGFRB‐Luc) spheroids were intraperitoneally injected into immunocompromised female mice so as to mimic the transcoelomic dissemination process of HGSOC. Animals injected with sh‐PDGFRB‐Luc aggregates presented a significantly lower number of metastatic lesions in comparison with those injected with sh‐CTRL‐Luc spheroids (Fig. 3F and Fig. S2E,F). Furthermore, all mice (*n* = 6) injected with sh‐CTRL‐Luc spheroids developed metastases in the omentum, which is the most frequent site for transcoelomic metastasis in HGSOC patients [34], but only four out of eight with sh‐PDGFRB‐Luc spheroids did (Fig. S2E,F). Altogether, our results demonstrate that ovarian cancer requires PDGFRβ for the formation of fully functional aggregates, which are able to disseminate through the peritoneal cavity and generate intra‐abdominal implants.

### 3.3 PDGFRβ‐induced fibronectin promotes ovarian cancer cell aggregation and cluster formation

**Fig. 4:**
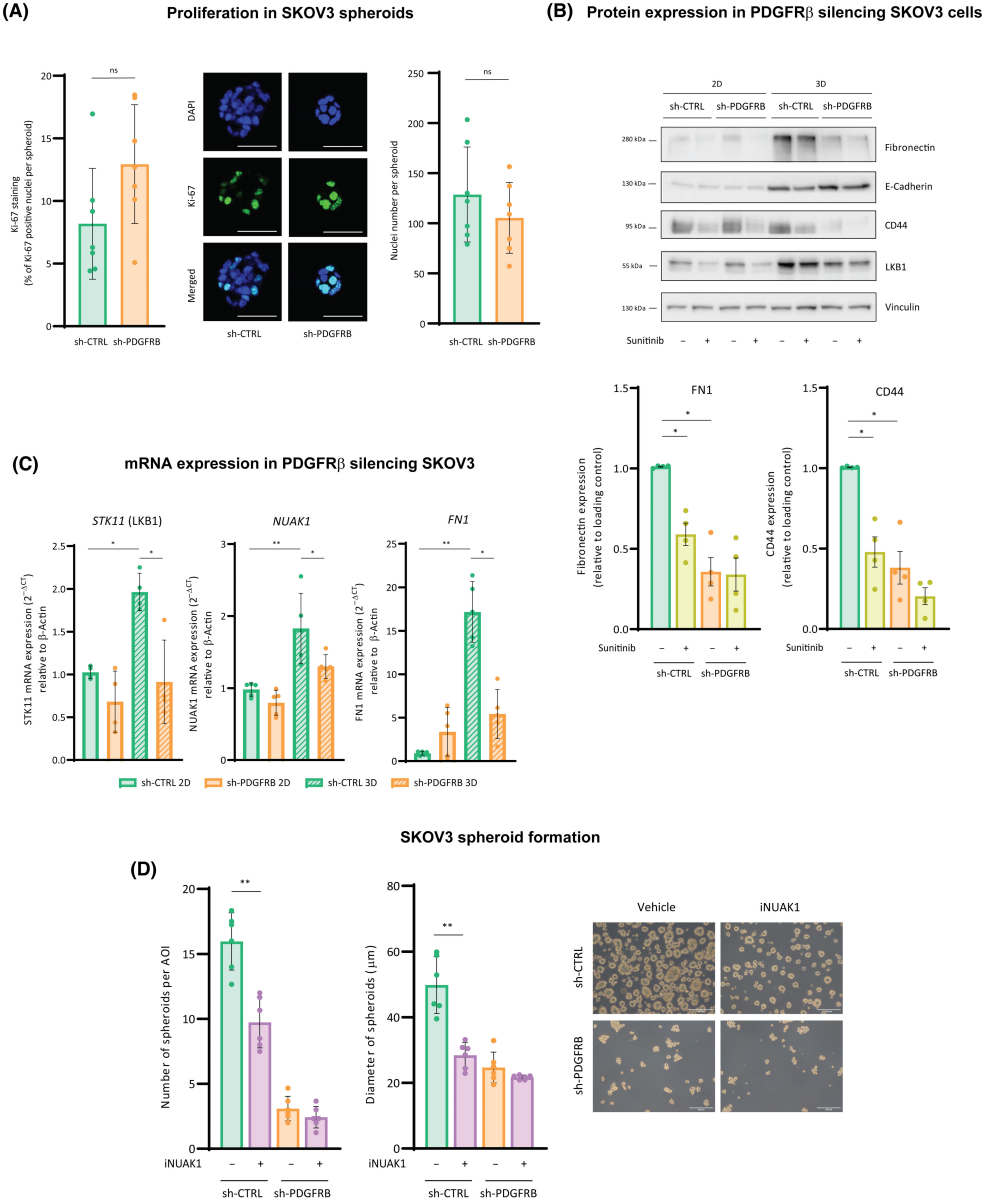
PDGFRβ‐induced fibronectin promotes ovarian cancer cell aggregation and cluster formation. (A) Proliferation of spheroids measured by Ki‐67 immunostaining (left panel) or nuclei number per spheroid (right panel). Middle panel: representative images of Ki67 staining in spheroids. (B) Western blot for fibronectin, E‐cadherin, CD44, and LKB1 expression in sh‐CTRL and sh‐PDGFRB cells upon 2D and 3D cultures and sunitinib treatment. Vinculin was used as loading control. A representative image is shown of four independent experiments. Lower panel: quantification of fibronectin and CD44 protein expression measured as in B. (C) mRNA expression of *STK11*, *NUAK1*, and *FN1* of sh‐CTRL and sh‐PDGFRB cells cultured in 2D and 3D conditions. (D) Number (left panel) and diameter (right panel) of spheroids generated from sh‐CTRL and sh‐PDGFRB cells upon iNUAK. Representative images are shown in A (out of *n* = 7 independent experiments, and 15 spheroid images per experimental condition) and D (out of *n* = 6 independent experiments, and 3 images per experimental condition). Scale bars mean 200 μm (D), and 50 μm (A). Data presented in A–D are shown Mean ± SD and each graph dot represents an independent experiment in A (*n* = 7), B (*n* = 4), C (*n* = 4 for *STK11* and *n* = 5 for *NUAK1* and *FN1*) and D (*n* = 6). Significant differences were assessed using Mann–Whitney *U*‐test when comparing between two groups and considered when *P* < 0.05 (**<0.01, *<0.05). When *P* > 0.05, differences were considered nonsignificative (ns).

We next aimed to investigate the molecular mechanism by which PDGFRβ was responsible for tumorsphere formation. We first examined whether PDGFRβ conferred an enhanced proliferative rate to ovarian cancer cells. Intriguingly, sh‐CTRL and sh‐PDGFRB spheroids presented similar Ki‐67 expression levels (10–15%) and similar nuclei number per spheroid (Fig. 4A). This observation suggested that spheroid proliferation was not involved on the pro‐tumorsphere formation effect mediated by PDGFRβ.

As ovarian cancer cells behave differently as spheroids than when cultured in adherent conditions, not only phenotypically but also at the molecular level (Fig. 4B and Fig. S3A), the expression of some epithelial‐to‐mesenchymal transition (EMT) markers was characterized in sh‐CTRL and sh‐PDGFRB spheroids (3D) and compared to those of adherent cells (2D). While E‐Cadherin, which was much more expressed in spheroids than in adherent cells, was higher in sh‐PDGFRB spheroids, CD44 and fibronectin expressions were observed to be lower in sh‐PDGFRB cells compared with sh‐CTRL ones (Fig. 4B and Fig. S3B). These results were replicated in sh‐CTRL and sh‐PDGFRB spheroids by pharmacological inhibition of PDGFRβ with sunitinib (Fig. 4B), suggesting that PDGFRβ expression was necessary for the expression of the stemness marker CD44 and the adhesion molecule fibronectin, especially in the 3D context, where the greatest differences were observed. Indeed, fibronectin was the one with the greatest increase in 3D compared with 2D that was also reduced in sh‐PDGFRB aggregates without the additional effect of sunitinib treatment. Fibronectin has been already involved in the generation of ovarian cancer spheroids through the activity of the kinases LKB1‐NUAK1 [35]. We postulated that PDGFRβ could be upstream of this axis during ovarian cancer cell aggregation. Fibronectin decrease upon PDGFRβ silencing was also observed at the mRNA level, along with those of *STK11* (LKB1) and *NUAK1* (Fig. 4C), indicating that all the axis LKB1‐NUAK1‐fibronectin was downregulated in absence of PDGFRβ. These observations were further validated *in silico* when *PDGFRB* expression was observed to positively correlate with those of *NUAK1* and *FN1* in HGSOC patients (Fig. S3B). Moreover, *PDGFRB* positively correlates too with three gene sets that include *FN1* (Table S5): ‘ECM‐receptor interaction’ and ‘focal adhesion’ gene signatures, which are the two top‐ranked predicted KEGG pathways for *PDGFRB* in the ARCHS^4^  database [36] (Table S6); and with ‘regulation of actin cytoskeleton’ gene signature, in primary tumors and metastases of HGSOC patients in all cases (Fig. S3C). Taken together, obtained data suggested that fibronectin expression in ovarian cancer is dependent on PDGFRβ and that it is responsible for cell aggregation. To confirm this, fibronectin expression was abolished by inhibiting NUAK1, and the number and diameter of sh‐CTRL spheroids generated were significantly reduced (Fig. 4D). In contrast, NUAKi had no effect on sh‐PDGFRB spheroids.

**Fig. 5:**
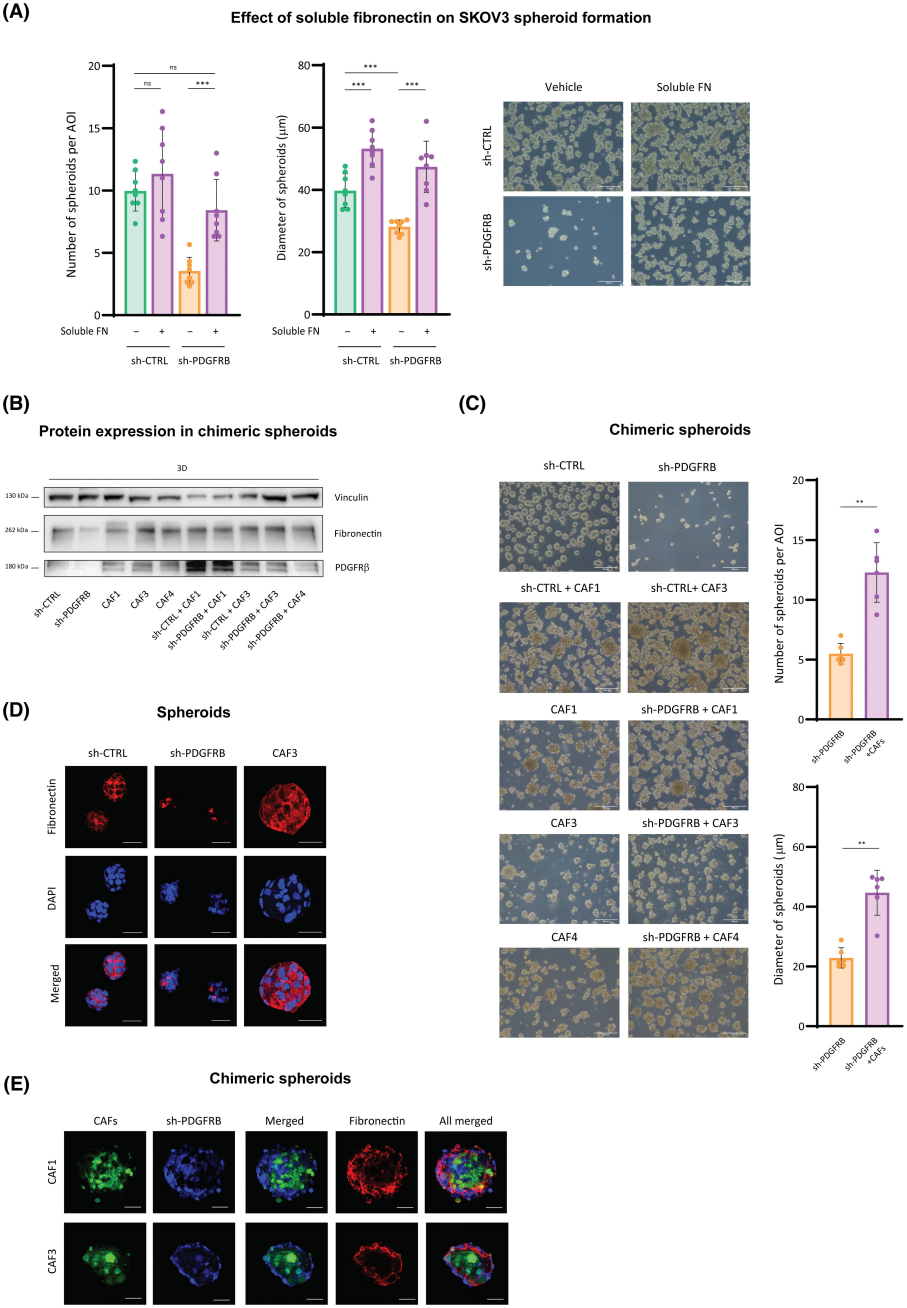
Fibronectin produced by CAFs recovers sh‐PDGFRB ovarian cancer cell aggregation and cluster formation. (A) Number (left panel) and diameter (right panel) of spheroids generated from sh‐CTRL and sh‐PDGFRB cells upon soluble fibronectin (FN) treatment. (B) Western blot for fibronectin and PDGFRβ expression in sh‐CTRL, sh‐PDGFRB and different CAF cell lines, cultured in 3D as spheroids and chimeric spheroids. Vinculin was used as loading control. A representative image of *n* = 3 independent experiments is shown. (C) Number (upper panel) and diameter (lower panel) of chimeric spheroids generated by 3D culture of sh‐CTRL and sh‐PDGFRB with different CAF cell lines. (D) Fibronectin immunostaining (red) in spheroids generated from sh‐CTRL, sh‐PDGFRB, and CAF3 cells. Nuclei were stained using DAPI (blue) (E) Fibronectin immunostaining (red) in chimeric spheroids generated by 3D coculture of sh‐PDGFRB (stained with Violet dye) with CAF cells (stained with green CFSE dye). Representative images of spheroids are shown in A (out of *n* = 8 independent experiments and 3 images per experimental condition) and C (out of *n* = 6 independent experiments and 3 images per experimental condition). Representative images of immunofluorescence on spheroids are shown in D and E (out of *n* = 3 independent experiments and 5 spheroid images per experimental condition). Scale bars mean 200 μm in A and C, and 50 μm in D and E. Data presented are shown mean ± SD, and each graph dot represents an independent experiment in A (*n* = 8) and C (*n* = 6). Significant differences were assessed using Mann–Whitney *U*‐test when comparing between two groups and considered when *P* < 0.05 (***<0.001, **<0.01). When *P* > 0.05, differences were considered nonsignificative (ns).

Conversely, when soluble fibronectin was added to sh‐PDGFRB cells when cultured in 3D conditions at the moment of cell seeding, it did recover sh‐CTRL spheroids phenotype, showing significant increases in number and diameter of spheroids generated (Fig. 5A). To further assess the involvement of PDGFRβ‐induced fibronectin in ovarian spheroids formation, fibronectin was provided to sh‐PDGFRB cells through 3D coculture with PDGFRβ–fibronectin‐expressing human CAFs (Fig. 5B). CAFs alone already generated spheroids in 3D conditions, which presented greater diameters when mixed with sh‐CTRL SKOV3 cells (Fig. 5C). Moreover, chimeric spheroids conformed by CAFs and sh‐PDGFRB cells did present cancer cells as well, increasing the number and diameter of those of sh‐PDGFRB, recovering sh‐CTRL spheroids phenotype (Fig. 5C). Next, we studied fibronectin distribution in spheroids by immunofluorescence. As expected, fibronectin was expressed at low levels in sh‐PDGFRB spheroids, compared with its high extracellular expression surrounding cells in sh‐CTRL and CAFs spheroids (Fig. 5D). Inside the chimeric spheroids, CAFs occupied the center of the spheroid, while tumor cells surrounded them on the periphery (Fig. 5E and Fig. S3C). Interestingly, fibronectin was observed to change the distribution presented in 3D cultured CAFs (Fig. 5D) and to be specifically localized at the limiting area between central CAFs and peripheral sh‐PDGFRB cells within the chimeric spheroids (Fig. 5E), which is indicative of its role in ovarian cancer cells adhesion. Collectively, all these data indicated that, regardless of its source, PDGFRβ‐induced fibronectin directs ovarian cancer cell aggregation and cluster formation.

**Fig. 6:**
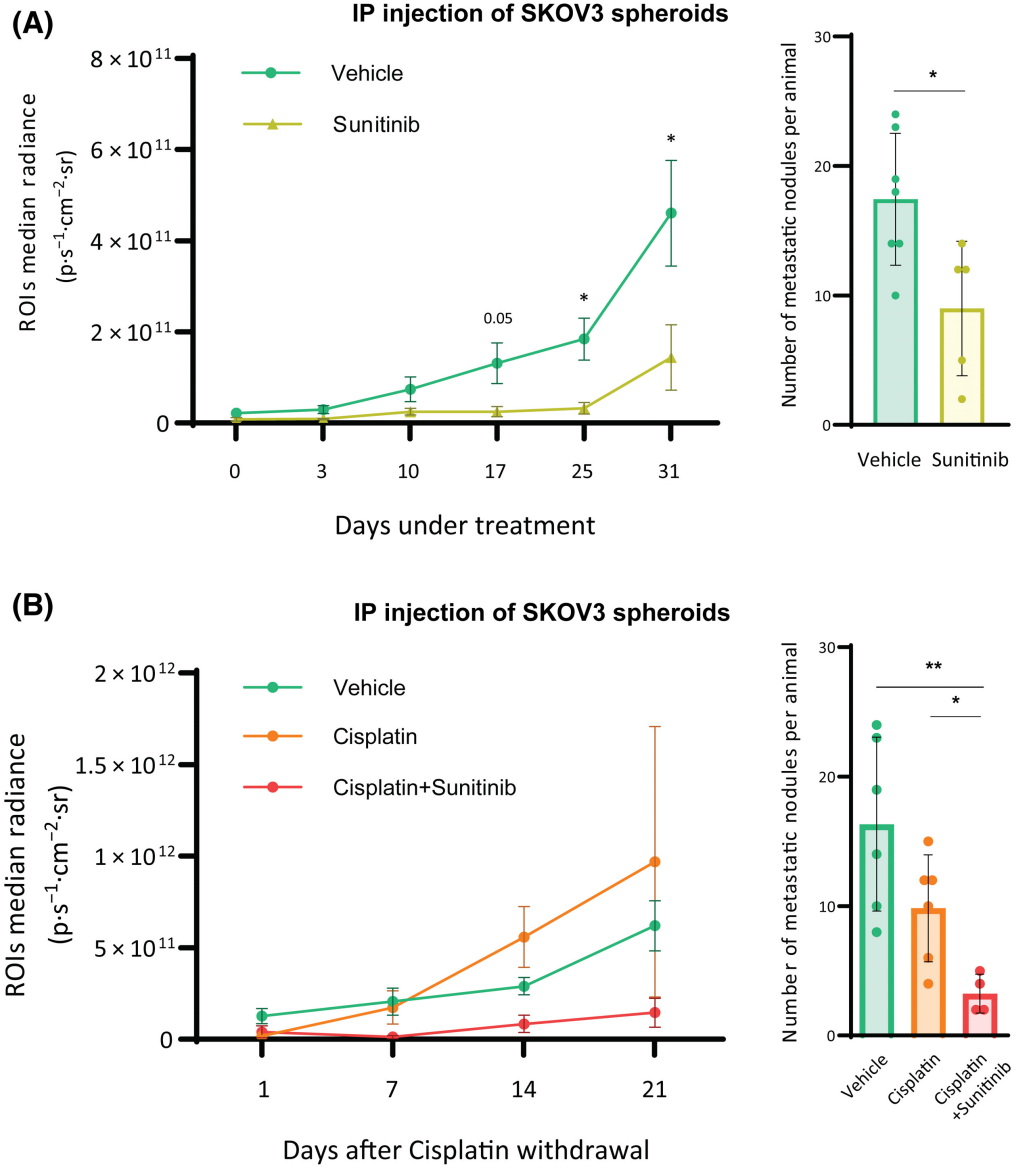
*In vivo* effect of sunitinib. (A) Left panel: ROIs radiance (expressed in p·s^−1^·cm^−2^·sr^−1^) of immunodeficient female mice intraperitoneally injected with 250 spheroids generated from sh‐CTRL‐Luc receiving vehicle (*n* = 7 mice) or Sunitinib (*n* = 5 mice). Data in this graph are shown mean ± SEM. Right panel: metastatic nodules per animal. (B) Left panel: ROIs radiance (expressed in p·s^−1^·cm^−2^·sr^−1^) of immunodeficient female mice intraperitoneally injected with 250 spheroids generated from sh‐CTRL‐Luc, having received vehicle (*n* = 6 mice), 2 doses of cisplatin alone (*n* = 6 mice) or 2 doses of cisplatin + daily sunitinib (*n* = 4 mice) at Day 1 after cisplatin withdrawal. From that point, mice were still treated with vehicle (vehicle group) or sunitinib (cisplatin + sunitinib group) for 21 additional days. Data in this graph are shown Mean ± SEM. Right panel: metastatic nodules per animal. Data presented are shown Mean ± SD unless otherwise specified, and each graph dot represents an independent animal. Significant differences were assessed using Mann–Whitney *U*‐test when comparing between two groups and considered when *P* < 0.05 (**<0.01, *<0.05).

Finally, in order to highlight the potential role of anti‐PDGFR pharmacological treatments as a therapeutic strategy to hamper the main metastatic route in HGSOC patients, two approaches were followed in which SKOV3 spheroids were intraperitoneally injected into immunodeficient female mice. Two weeks later, when disseminations had already been generated, animals were treated. In a first experiment, sunitinib was daily administered. After 1 month of treatment, sunitinib prevented disseminations growth in treated animals, which also presented a significantly lower number of metastatic nodules compared to those receiving vehicle (Fig. 6A). In a second experiment, we sought to evaluate the effect of sunitinib as maintenance therapy, along with the standard cisplatin treatment and after cisplatin withdrawal. Thus, three experimental groups were set. The first one received one dose of cisplatin alone per week only for the first 2 weeks (cisplatin group) and was administered vehicle thereafter. The second group was administered also cisplatin in the same posology, and at the same time and throughout the following 21 days, received sunitinib daily (cisplatin+sunitinib group). The third group received vehicle alone throughout all the experiment (vehicle group). Cisplatin was initially very effective, impairing the growth of tumor masses (Day 1 after cisplatin withdrawal), but metastases eventually regrew in the absence of cisplatin, reaching even higher values than the vehicle group (Fig. 6B). In contrast, the sunitinib pressure prevented disseminations regrowth as well as it decreased the presence of metastatic nodules (70% lower) compared to those receiving vehicle, or even cisplatin alone. Overall, our results confirmed not only that PDGFRβ is key for ovarian tumorsphere formation but also that it could be a potential therapeutic target to avoid tumorsphere‐directed metastatic spread in HGSOC, if combined with the standard of care cisplatin.

## 4 Discussion

Transcoelomic dissemination is the most frequent and aggressive route for tumor spread in HGSOC, and most patients eventually die from abdominal complications [2, 3, 37, 38]. However, the molecular mechanisms that direct tumorsphere formation and proliferation in the ascitic liquid are poorly known, and a better understanding of them will provide us with tools to block ovarian cancer progression and metastasis [2, 28, 29, 30, 31, 32]. In the present study, we identify PDGFRβ as responsible for NUAK1‐fibronectin‐induced cellular clustering and the resulting tumorsphere formation.

We show that sunitinib or pazopanib treatment dramatically decreases number and size of ovarian tumorspheres isolated from ascitic liquid from HGSOC patients, increasing the number of isolated cells, which makes them more susceptible to undergo anoikis cell death. Similarly, ovarian cancer cells cultured in 3D conditions are unable to generate spheroids upon sunitinib treatment, and neither do the ones that have PDGFRβ silenced by shRNA. Hence, it stands to reason that PDGFRβ is responsible for ovarian cancer cell clustering. Indeed, PDGFRβ has been described to be expressed in tumor cells as well as in the stromal component of ovarian carcinomas [39, 40]. Our results confirm that primary tumors that induce ascites accumulation, which in turn contain ovarian cancer cells and tumorspheres, express high levels of PDGFRβ. Moreover, those tumorspheres that present increased expressions of PDGFRβ are provided with an enhanced metastatic potential, which is evidenced by higher expression of PDGFRβ in HGSOC implants compared with primary tumors reported here. In fact, CAFs have been described to recruit ovarian cancer cells to generate cellular aggregates [17] by PDGF‐BB secretion and stimulation of PDGFRβ expression in ovarian cancer cells [41]. If we block PDGFRβ activity by using sunitinib, the metastatic capacity of spheroids decreases. Consequently, according to our data, those patients that present high PDGFRβ levels in ovarian primary tumors could be suitable candidates for receiving inhibitors such as sunitinib or pazopanib. Therefore, PDGFR inhibitors as adjuvant treatment after debulking surgery could be a potential therapeutic strategy to avoid intra‐abdominal recurrence in HGSOC patients, even after having responded to initial therapy.

Our results indicate that PDGFRβ exerts a pro‐adhesive function through the activation of fibronectin in cancer cells. The PDGFRs‐fibronectin axis has been described to be active in mesenchymal stromal stem cells (MSCs), where it has a mesenchyme‐promoting role. By contrast, when PDGFRs are inhibited, MSCs have been reported to acquire an epithelial phenotype and upregulate E‐Cadherin [42]. Our results indicate that this process also takes place in ovarian cancer cells. When PDGFRβ is pharmacologically inhibited or genetically silenced in 3D conditions, E‐cadherin expression is increased and cells lose their ability to aggregate and adhere to the substrate instead. Interestingly, we observed fibronectin expression levels to be minimal in those few, successfully generated sh‐PDGFRB aggregates, which we had proven to exhibit an impaired disseminative capacity *in vivo*. Hence, our data indicate that PDGFRβ‐directed fibronectin expression in ovarian cancer cells allows them to aggregate with each other and generate compact, disseminative‐efficient tumorspheres. Moreover, our results also place the reported LKB1‐NUAK1‐fibronectin pathway in ovarian cancer spheroids [35] below PDGFRβ. Fibronectin has also been reported to be necessary for integrin‐mediated interaction between cancer cells, contributing to ovarian cancer spheroid compaction and survival to anoikis cell death [43], and it has been found to be elevated in the ascitic liquid from ovarian cancer patients [44, 45]. Interestingly, we demonstrate here that in the absence of PDGFRβ in ovarian cancer cells, PDGFRβ‐expressing CAFs could provide fibronectin to sh‐PDGFRB cells to generate chimeric spheroids. This probably takes place in these tumors that present low PDGFRβ expression. Overall, we demonstrate that, regardless of its source, PDGFRβ‐induced fibronectin not only directs initial cell clustering but also maintains ovarian cancer cells together, allowing their survival within the ascitic fluid.

## 5 Conclusions

Conclusively, this work sheds light on the molecular mechanism that directs tumorsphere formation, which is critical for HGSOC metastatic spread and is governed by PDGFRβ. The preclinical findings presented here may represent an opportunity to turn the main ovarian cancer's metastatic potential promoters, tumorspheres, into this cancer's Achilles' heel.

## Conflict of interest

The authors declare no conflict of interest.

## Author contributions

NG‐S and FV contributed to conception and design. NG‐S, AF, KG, AL, EA‐S, JAM‐J, AV, XM‐G, SF‐G, MB, LM, and JP contributed to acquisition of data (provided animals, acquired and managed patients, provided facilities, etc.). NG‐S contributed to analysis and interpretation of data (e.g., statistical analysis, biostatistics, and computational analysis). NG‐S, JAM‐J, JP, and FV contributed to writing, review, and/or revision of the manuscript. FV contributed to study supervision.

### Peer review

The peer review history for this article is available at https://www.webofscience.com/api/gateway/wos/peer‐review/10.1002/1878‐0261.13556.

## Supporting information


**Fig. S1.** PDGFRβ is increased in HGSOC primary tumors and even so in metastatic lesions.

**Fig. S2.** PDGFRβ plays a role in ovarian cancer spheroid formation and in the disseminative capacity of ovarian tumor cells during metastatic spread.

**Fig. S3.** PDGFRβ‐induced fibronectin promotes ovarian cancer cell aggregation and cluster formation.

**Table S1.** HGSOC cohort of patients.
**Table S2.** Factors and drugs used for cell, spheroid, and tumorsphere treatment.
**Table S3.** Primers used for real‐time PCR.
**Table S4.** Primary antibodies used for western blot.
**Table S5.** Gene signatures studied.
**Table S6.** Top 10 predicted KEGG pathways for *PDGFRB* in the ARCHS^4^ database [36].

## Data Availability

This study has used data from two databases publicly available in Gene Expression Omnibus (GEO accession numbers GSE14407 and GSE137237, respectively). The rest of data that support our findings, as well as reagents and cell lines that have been developed throughout our study, will be made available to the scientific community from the corresponding author (fvinals@ub.edu) upon reasonable request.
