# Supplementary material for: Ovarian cancer relies on the PDGFRβ–fibronectin axis for tumorsphere formation and metastatic spread
Source: Mol Oncol. 2023 Dec 1;18(1):136–55. doi: 10.1002/1878-0261.13556 (PMC10766197; doi:10.1002/1878-0261.13556)
Supplement: Supplementary file 4 — Table S1. HGSOC cohort of patients. Table S2. Factors and drugs used for cell, spheroid, and tumorsphere treatment. Table S3. Primers used for real‐time PCR. Table S4. Primary antibodies used for western blot. Table S5. Gene signatures studied. Table S6. Top 10 predicted KEGG pathways for PDGFRB in the ARCHS4 database [36]. [file MOL2-18-136-s001.docx]

**SUPPLEMENTARY FIGURES**

**Supplementary Figure 1. PDGFRβ is increased in HGSOC primary tumors and even so in metastatic lesions. A** Overview of HGSOC patients cohort. *BRCA1/2* mutational status, age, FIGO stage and samples available per patient are depicted. WT: wild-type; MUT: mutated; Prim: primary tumor; Met: metastases; Asc: ascites. **B** Protein expression of sunitinib targets in primary tumors paired with ascites samples. Representative images (out of n=14 tissue samples and 3 images per sample) are shown, and scale bars mean 200μm. **C** and **D** RNA expression of *PDGFRB* in primary tumors and metastases from publicly available data in Gene Expression Omnibus (GSE137237) (**C**) and early and advanced tumors (GSE14407) (**D**). Data presented are shown Mean ± SD and each graph dot represents an independent patient in **C** (n=11 patients per group) and **D** (n=4 patients in Early group and n=8 patients in Advanced group). Significant differences were assessed using Mann-Whitney U-test when comparing between two groups, and considered when P<0.05 (*<0.05, ****<0.0001).

**Supplementary Figure 2. PDGFRβ plays a role in ovarian cancer spheroid formation and in the disseminative capacity of ovarian tumor cells during metastatic spread. A** Western blot for PDGFRβ expression in dox-sh-CTRL cells and dox-sh-PDGFRB clones 1 and 2 after doxycycline stimulation (DOX) for 24 hours. A representative image of n=3 independent experiments is shown. **B** Adhered dox-sh-PDGFRB1 cells upon DOX stimulation. Representative images (out of n=3 independent experiments and 3 images per experimental condition) are shown. **C** Measurement of ROIs radiance (expressed in p/sec/cm^2^/sr) of omental metastases generated after intraperitoneal injection of 500.000 dox-sh-PDGFRB1 cells into immunodeficient female mice with (+DOX, n=4 mice) or without (-DOX, n=4 mice) DOX. Data in this graph are presented Mean ± SEM. **D** Anoikis cell death by PI staining of A2780 spheroids upon sunitinib treatment. Representative images (out of n=4 independent experiments and 3 images per experimental condition) are shown. **E** and **F** Metastatic nodules per animal (**E**) and % of animals presenting metastatic nodules (**F**) after IP injection of 250 sh-CTRL-Luc or sh-PDGFRB-Luc spheroids. Data presented are shown Mean ± SD unless otherwise specified, and each graph dot represents an independent experiment in **D** (n=4) and an independent animal in **C** (n=4 mice per group) and **E** (n=6 mice per group). Scale bars mean 20µm in **B** and 200µm in **D**. Significant differences were assessed using Mann-Whitney U-test when comparing between two groups, and considered when P<0.05 (*<0.05).

**Supplementary Figure 3. PDGFRβ-induced fibronectin promotes ovarian cancer cell aggregation and cluster formation. A** Western blot for the expression of fibronectin, E-Cadherin, CD44, β-Catenin and Vimentin in SKOV3 cells cultured in 2D and 3D conditions. Vinculin was used as loading control. A representative image of n=3 independent experiments is shown. **B** Correlation of *PDGFRB* expression with *NUAK1* and *FN1* in HGSOC patients (n=11 patients per group). **C** Correlation of *PDGFRB* RNA expression with gene signatures including *FN1* in HGSOC patients (n=11 patients per group). Pearson’s correlation was used for calculating R and P values. Significant differences were considered when P<0.05. **D** Chimeric spheroids obtained by coculture of CAF1-4 cell lines (stained in green with CFSE dye) with sh-PDGFRB cells (stained with Violet dye) in 3D conditions. Nuclei are stained with 7-AAD Red. Representative images (out of n=3 independent experiments, and 5 spheroid images per experimental condition) are shown, and scale bars mean 50µm.

**SUPPLEMENTARY TABLES**

**Supplementary Table 1. HGSOC cohort of patients.** Clinical data from HGSOC treatment-naïve patients studied. Age, ovarian cancer subtype (AP), FIGO stage (FIGO), *BRCA* mutational status (*BRCA1/2*),samples available for study (Samples available) and Metastatic site (Metastatic site) are shown. UNK: unknown; No mut: no mutation; *BRCA1*: *BRCA1* mutated; *BRCA2*: *BRCA2* mutated; Prim: primary tumor; Met: metastases; Asc/ASC: ascites; OV: ovary; OM: omentum; ADP: adipose tissue; PT: peritoneum.

| **Patient** | **Age** | **AP** | **FIGO** | ***BRCA1/2*** | **Samples available** | **Metastatic site** |
| --- | --- | --- | --- | --- | --- | --- |
| 01 | 56 | HGSOC | IIIC | UNK | Prim | UNK |
| 02 | 45 | HGSOC | IIIC | *BRCA2* | Prim, Met | OV, OM |
| 03 | 67 | HGSOC | IIIC | UNK | Prim, Met | OV, OM |
| 04 | 58 | HGSOC | IIIC | No mut | Prim, Met | OV, ADP |
| 05 | 51 | HGSOC | IIIC | *BRCA2* | Prim, Met | OV, OM |
| 06 | 77 | HGSOC | IIIC | UNK | Prim, Met | OV, PT |
| 07 | 49 | HGSOC | IIIC | No mut | Prim, Met | OV, OM |
| 08 | 44 | HGSOC | IIIC | No mut | Prim, Met | OV, OM |
| 09 | 62 | HGSOC | IIIC | No mut | Prim, Met | OV, OM |
| 10 | 69 | HGSOC | IIIC | No mut | Prim | OV |
| 11 | 68 | HGSOC | IIIC | *BRCA2* | Prim | OV |
| 12 | 54 | HGSOC | IIIC | *BRCA1* | Prim | OV |
| 13 | 65 | HGSOC | IC | No mut | Prim | OV |
| 14 | 50 | HGSOC | IC | No mut | Prim | OV |
| 15 | 76 | HGSOC | IIIB | UNK | Met | OM |
| 16 | 63 | HGSOC | IIIC | No mut | Met | UNK |
| 17 | 37 | HGSOC | IIIC | UNK | Met | OM |
| 18 | 39 | HGSOC | IIIC | UNK | Asc | ASC |
| 19 | 63 | HGSOC | IIIC | No mut | Asc | ASC |
| 20 | 66 | HGSOC | IVA | UNK | Prim, Asc | OV, ASC |
| 21 | 49 | HGSOC | IIIC | No mut | Prim, Met, Asc | OV, OM, ASC |
| 22 | 66 | HGSOC | IIIC | UNK | Prim, Met, Asc | UNK, OM, ASC |
| 23 | 48 | HGSOC | IVA | *BRCA1* | Prim, Asc | OV,ASC |
| 24 | 80 | HGSOC | IVA | No mut | Asc | ASC |
| 25 | 73 | HGSOC | IVB | No mut | Prim, Asc | OV,ASC |
| 26 | 73 | HGSOC | IVA | No mut | Prim, Asc | OV,ASC |
| 27 | 43 | HGSOC | IIIC | No mut | Asc | ASC |
| 28 | 50 | HGSOC | IVA | *BRCA2* | Asc | ASC |
| 29 | 83 | HGSOC | IVB | No mut | Asc | ASC |
| 30 | 73 | HGSOC | IVA | No mut | Asc | ASC |
| 31 | 75 | HGSOC | IIIC | UNK | Asc | ASC |
| 32 | 69 | HGSOC | IVB | *BRCA2* | Asc | ASC |

**Supplementary Table 2. Factors and drugs used for cell, spheroid and tumorsphere treatment**

| **Factor or drug** | **Dose used** | **Reference** |
| --- | --- | --- |
| Cisplatin | 10µM | Accord, Cat. Num. 683048.2OH |
| Gefitinib (ZD1839) | 1µM | Selleckchem, Cat. Num. S1025 |
| Lapatinib | 1µM | Kindly provided by GlaxoSmithKline |
| iNUAK1 (HTH-01-015) | 100nM | MedChemExpress, Cat. Num. #HY-12334 |
| Pazopanib | 1µg/mL | Kindly provided by GlaxoSmithKline |
| Soluble fibronectin | 5µg/mL | Sigma-Aldrich, Cat. Num. #F2006 |
| Sunitinib (SU 11248) | 15µM | MedChemExpress, Cat. Num. #HY-10255A |
| TGFβRi (LY2109761) | 2µM | Kindly provided by Eli Lilly |

**Supplementary Table 3. Primers used for Real-Time PCR**

| **Gene** | **Forward primer** | **Reverse primer** |
| --- | --- | --- |
| FN1 | AGGAAGCCGAGGTTTTAACTG | AGGACGCTCATAAGTGTCACC |
| KIT | TGGATCTATATGAACAGAACCTTCA | GCTTTCATTCTCAGACTTGGG |
| NUAK1 | TTACATCAGTGAGCGGCGAC | TGGACCACACCGTTCTTGTG |
| PDGF-AA | TACTGAATTTCGCCGCCACA | CCAAAGAATCCTCACTCCCTACG |
| PDGF-BB | TGTGCGGAAGAAGCCAATCT | AATAACCCTGCCCACACACTC |
| PDGFRA | TGTAACTGGCGGATTCGAG | ATAAAGTGGTTTTCTGAACGGG |
| PDGFRB | CATCACCGTGGTTGAGAGC | AATTGTAGTGTGCCCACCTCTC |
| STK11 | GGCTCTTACGGCAAGGTGAA | CTCCTCAGTAGTTGAATTTCCTTCT |
| VEGFR2 | TGTACGGTCTATGCCATTCCT | GGGTATGGGTTTGTCACTGAG |

**Supplementary Table 4. Primary Antibodies used for Western Blot**

| **Protein** | **Species** | **Dilution** | **Reference** |
| --- | --- | --- | --- |
| β-Catenin | Mouse | 1:1000 | BD Transduction Laboratories, Cat. Num. #610153 |
| CD44 | Mouse mAb | 1:1000 | Cell Signaling, Cat. Num. #3570 |
| E-Cadherin | Rabbit mAb | 1:1000 | Cell Signaling, Cat. Num. #3195 |
| Fibronectin | Rabbit pAb | 1:1000 | Abcam, Cat. Num. #ab2413 |
| LKB1 | Mouse mAb | 1:1000 | Santa Cruz Biotechnology, Cat. Num. #sc-32245 |
| PDGFRβ | Rabbit pAb | 1:1000 | Santa Cruz Biotechnology, Cat. Num. #sc-339 |
| Tubulin | Mouse mAb | 1:2500 | Sigma-Aldrich, Cat. Num. #T6199 |
| Vimentin | Mouse mAb | 1:1000 | ThermoFisher Scientific, Cat. Num. #MA5-11883 |
| Vinculin | Mouse | 1:2500 | Sigma-Aldrich, Cat. Num. #V9264 |

**Supplementary Table 5. Gene signatures studied**

| **Gene Set** | **Standard name** | **Systematic name** | **Database** |
| --- | --- | --- | --- |
| ECM-receptor interaction | KEGG_ECM_RECEPTOR_INTERACTION | M7098 | MSigDB |
| Focal adhesion | KEGG_FOCAL_ADHESION | M7253 | MSigDB |
| Regulation of actin cytoskeleton | KEGG_REGULATION_OF_ACTIN_CYTOSKELETON | M18306 | MSigDB |

**Supplementary Table 6. Top 10 predicted KEGG pathways for *PDGFRB* in the ARCHS^4^**^35^ **database**

| **Rank** | **Gene Set** | **Z-score** |
| --- | --- | --- |
| 1 | ECM-receptor interaction_Homo sapiens_hsa04512 | 4.71930319 |
| 2 | Focal adhesion_Homo sapiens_hsa04510 | 3.39180769 |
| 3 | Dilated cardiomyopathy_Homo sapiens_hsa05414 | 2.92269968 |
| 4 | Hypertrophic cardiomyopathy (HCM)_Homo sapiens_hsa05410 | 2.84136513 |
| 5 | Protein digestion and absorption_Homo sapiens_hsa04974 | 2.837251 |
| 6 | Arrhythmogenic right ventricular cardiomyopathy (ARVC)_Homo sapiens_hsa05412 | 2.64366374 |
| 7 | Amoebiasis_Homo sapiens_hsa05146 | 2.37421857 |
| 8 | AGE-RAGE signaling pathway in diabetic complications_Homo sapiens_hsa04933 | 2.33743326 |
| 9 | Proteoglycans in cancer_Homo sapiens_hsa05205 | 2.21324135 |
| 10 | Glycosaminoglycan biosynthesis - chondroitin sulfate / dermatan sulfate_Homo sapiens_hsa00532 | 2.03894695 |
